# Heterostructure Engineering of 2D Superlattice Materials for Electrocatalysis

**DOI:** 10.1002/advs.202204297

**Published:** 2022-10-20

**Authors:** Zhen Zhang, Peizhi Liu, Yanhui Song, Ying Hou, Bingshe Xu, Ting Liao, Haixia Zhang, Junjie Guo, Ziqi Sun

**Affiliations:** ^1^ Key Laboratory of Interface Science and Engineering in Advanced Materials Ministry of Education Taiyuan University of Technology Taiyuan 030024 P. R. China; ^2^ Materials Institute of Atomic and Molecular Science Shaanxi University of Science & Technology Xi'an 710021 P. R. China; ^3^ School of Mechanical Medical and Process Engineering Queensland University of Technology Brisbane QLD 4000 Australia; ^4^ School of Chemistry and Physics Queensland University of Technology Brisbane QLD 4000 Australia

**Keywords:** 2D superlattice materials, electrocatalysis, hydrogen evolution reaction, oxygen evolution reaction, oxygen reduction reaction

## Abstract

Exploring low‐cost and high‐efficient electrocatalyst is an exigent task in developing novel sustainable energy conversion systems, such as fuel cells and electrocatalytic fuel generations. 2D materials, specifically 2D superlattice materials focused here, featured highly accessible active areas, high density of active sites, and high compatibility with property‐complementary materials to form heterostructures with desired synergetic effects, have demonstrated to be promising electrocatalysts for boosting the performance of sustainable energy conversion and storage devices. Nevertheless, the reaction kinetics, and in particular, the functional mechanisms of the 2D superlattice‐based catalysts yet remain ambiguous. In this review, based on the recent progress of 2D superlattice materials in electrocatalysis applications, the rational design and fabrication of 2D superlattices are first summarized and the application of 2D superlattices in electrocatalysis is then specifically discussed. Finally, perspectives on the current challenges and the strategies for the future design of 2D superlattice materials are outlined. This review attempts to establish an intrinsic correlation between the 2D superlattice heterostructures and the catalytic properties, so as to provide some insights into developing high‐performance electrocatalysts for next‐generation sustainable energy conversion and storage.

## Graphical abstract

2D superlattice materials combining the advantages of 2D materials and advanced composites provide tunable physical and chemical properties to meet diverse requirements in different applications. This review article summarizes the major fabrication methods for preparing 2D superlattice materials and reviews their applications in electrocatalysis involved in emerging energy devices.

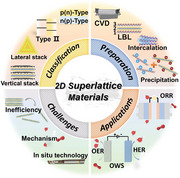

## 1 Introduction

To support the sustainable development of our society, it is an urgent priority to innovate clean energy devices based on green fuel resources.^[^ ^1^ ^]^ Hydrogen energy is a very promising energy with zero carbon dioxide emission, which has brought great progress in sustainable hydrogen technologies, such as electrochemical water splitting for hydrogen generation^[^ ^2^, ^3^, ^4^, ^5^, ^6^, ^7^ ^]^ and hydrogen‐based energy devices, including fuel cells.^[^ ^1^, ^6^, ^8^ ^]^ The electrochemical generation and application of hydrogen, however, have been significantly limited by the absence of low‐cost but effective catalysts. At present, noble metal‐based catalysts, i.e., Pt, Ir, Ru, etc., are still the most efficient catalysts in hydrogen‐based fuel generation and energy devices.^[^ ^9^, ^10^, ^11^, ^12^, ^13^ ^]^ The exploration of low‐cost, stable, and efficient nonnoble metal‐based catalysts, therefore, is one critical key to unlock the industrial applications of hydrogen energy with affordable cost.^[^ ^14^, ^15^, ^16^, ^17^, ^18^, ^19^, ^20^, ^21^ ^]^

The success of graphene with fascinating chemical and physical properties, such as large specific surface area and ultra‐high carrier mobility, has opened the door into 2D materials.^[^ ^22^, ^23^, ^24^, ^25^, ^26^, ^27^, ^28^ ^]^ Plenty of interesting 2D materials with unique physicochemical properties, such as Group IV–V element monolayers,^[^ ^29^, ^30^, ^31^, ^32^, ^33^, ^34^ ^]^ layered double hydroxides (LDHs),^[^ ^19^, ^35^, ^36^, ^37^, ^38^, ^39^, ^40^, ^41^ ^]^ transition metal oxides (TMOs),^[^ ^42^, ^43^, ^44^, ^45^, ^46^, ^47^, ^48^ ^]^ transition metal dichalcogenides (TMDs),^[^ ^49^, ^50^, ^51^, ^52^, ^53^ ^]^ MXens,^[^ ^44^, ^54^, ^55^, ^56^, ^57^, ^58^, ^59^, ^60^, ^61^ ^]^ hexagonal boron nitride (h‐BN),^[^ ^62^, ^63^, ^64^, ^65^, ^66^ ^]^ etc., have been discovered. These novel 2D materials have presented unparalleled performance to their corresponding bulk materials in the applications of energy conversion, harvesting, and conversion.^[^ ^38^, ^67^, ^68^, ^69^, ^70^, ^71^, ^72^ ^]^ Despite the unique performance, the pristine 2D materials are unable to meet the diverse requirements raised by the specific demands of the various energy technologies.^[^ ^73^, ^74^, ^75^, ^76^ ^]^ Fortunately, 2D materials provide a great platform to build heterostructures with other property‐complementary materials.^[^ ^7^, ^63^, ^77^, ^78^, ^79^, ^80^, ^81^, ^82^, ^83^, ^84^, ^85^, ^86^, ^87^, ^88^, ^89^ ^]^ Particularly, 2D materials usually have strong ionic or covalent bonding between the in‐plain atoms but have weak interlayer interactions, such as by van der Waals (vdW) forces, which allows the formation of layered heterostructures via vdW assembly or hetero‐atom intercalation. As we expected, the 2D‐based heterostructures indeed provided much improved properties and enhanced the performance of sustainable energy and environmental devices.^[^ ^90^, ^91^, ^92^, ^93^, ^94^, ^95^, ^96^, ^97^, ^98^ ^]^

To date, a variety of 2D‐based heterostructures have been discovered by combining the 2D materials with the decoration materials at dimensionalities of 1D, 2D, or 3D. Among them, 2D superlattice materials, a type of unique heterostructures composed of two different 2D monolayer or few‐layer nanosheets (A and B) in an alternate stacking sequence (ABABABAB…), have been regarded as an emerging family of 2D heterogenous materials.^[^ ^79^, ^80^, ^81^, ^99^, ^100^, ^101^, ^102^, ^103^, ^104^, ^105^, ^106^, ^107^ ^]^ Different from other 2D/2D heterostructures which are actually a sort of 2D nanocomposites with disordered stacks of AAABABBA…, the 2D superlattices possess strictly ordered stacking sequences and can provide the materials with more tailorable physicochemical properties, such as the ultrafast interlayer mass and charge transfer properties and the electronic tuning effect.^[^ ^11^, ^77^, ^108^, ^109^ ^]^ In general, 2D superlattice materials with molecular scale ordering and tunable interlayer interactions can possess some specific advantages: i) the strongly coupled interfaces between the two constructing layers can give optimized electronic configuration and promoted chemical or electrochemical activity;^[^ ^79^, ^80^, ^81^, ^99^, ^100^, ^101^, ^110^ ^]^ ii) the coexistence of two dissimilar materials, such as Janus structures, can provide desired combinations of chemical and physical properties;^[^ ^79^, ^81^, ^101^ ^]^ iii) via the selection of proper coupling constructing layers, specific chemically active surfaces, sites, or interfaces with proper intermediates adsorption behaviors for catalysis reactions can be effectively constructed.^[^ ^81^, ^100^, ^101^, ^111^ ^]^ For example, in a 2D superlattice of MoS_2_/LDHs, where the LDHs were coated by MoS_2_ nanosheets, the acid corrosion of LDHs in the acid medium was effectively suppressed by the protection of MoS_2_ monolayers and the electrocatalytic performance and stability were significantly improved.^[^ ^81^, ^100^ ^]^ Even though plausive progress in the theoretical and experimental studies of 2D superlattice materials has been achieved and few excellent reviews on their fabrication have been published,^[^ ^112^, ^113^, ^114^ ^]^ the construction of 2D superlattice materials for electrocatalysis applications has rarely been reviewed to date.

**Figure 1:**
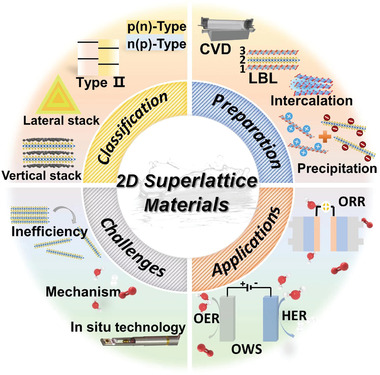
Graphic overview of the classification, preparation, electrocatalysis applications, and challenges of 2D superlattice materials.

Herein, we summarize the recent advances in the construction of 2D superlattices by using different 2D starting nanomaterials and their typical applications in electrocatalysis for sustainable fuel generations. First, the major fabrication methods for representative 2D superlattice materials are summarized. Then, the applications of 2D superlattice materials in electrocatalysis, including hydrogen evolution reaction (HER), oxygen evolution reaction (OER), overall water splitting (OWS), and oxygen reduction reaction (ORR), are discussed. Furthermore, the reaction kinetics and the fundamental mechanisms in the electrocatalysis of 2D superlattice catalysts are systematically reviewed. At the end, the current challenges and the prospective of this research topic are outlined. **Figure** **1** displays the graphic overview of this critical review on 2D superlattice materials.

## 2 Classifications of 2D Superlattice Materials

The emergence of superlattice materials is of great significance, which greatly expands the members of 2D materials. Artificial superlattice materials provide great potential to tailor the physicochemical properties of materials via crystal lattice matching or mismatching, interfacial coupling, electronic band structure aligning, mass and charge transport modulating, light responding and exiting, etc., of the periodic stacking of two different layered structures.^[^ ^102^, ^112^, ^115^, ^116^, ^117^ ^]^ To date, 2D superlattice materials have become one of the most attractive model materials in condensed matter physics and energy science, due to their specific surface and interface characteristics and their unique optical, electrical, thermal, and mechanical properties.^[^ ^78^, ^82^, ^105^, ^111^, ^112^, ^113^, ^114^, ^118^, ^119^, ^120^ ^]^ To better understand and use the 2D superlattice materials, this family materials can be classified into different categories in terms of the staking manners, the band alignment modes, the semiconductor types, and the constitutional compositions.

### 2.1 2D Superlattice Materials with Different Stacking Manners

**Figure 2:**
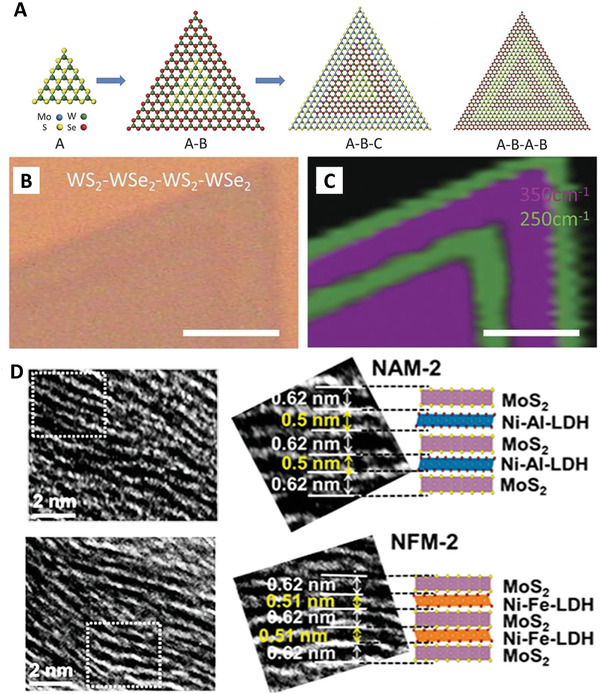
Laterally stacked superlattice and vertically stacked superlattice materials. A) A typical growth process of laterally stacked 2D superlattice materials. B) Optical microscope image of a laterally stacked 2D WS_2_‐WSe_2_ superlattice. C) Raman mapping image of 2D WS_2_‐WSe_2_ superlattice. Scale bars, 5 µm. Reproduced with permission.^[^ ^106^ ^]^ Copyright 2017, American Association for the Advancement of Science. D) High‐resolution transmission electron microscopy (HRTEM) and model diagrams of vertically stacked 2D MoS_2_‐LHD superlattice. Reproduced with permission.^[^ ^81^ ^]^ Copyright 2018, American Chemical Society.

Based on the stacking manners, 2D superlattice materials are categorized as vertically stacked superlattice and laterally stacked superlattice. **Figure** **2**A–C presents one typical laterally stacked superlattice, which consists of alternate epitaxial growth of 2D A structure surrounding a 2D B laterally within the same plane.^[^ ^106^ ^]^ The vertically stacked superlattice, as shown in Figure 2D, presents the structure of 2D superlattice in a periodic vertically stacking manner.^[^ ^81^ ^]^ Via the proper choose of the alignment of the constitutional 2D materials, the band structure of the 2D superlattice materials can be effectively controlled. By adjusting the compositions and stacking manners, the 2D superlattice materials with tailored band alignment modes have demonstrated broad application prospects in several fields, such as tunnel transistors, light‐emitting diodes, photodetectors, electrocatalysis, etc.^[^ ^107^, ^118^, ^121^, ^122^ ^]^

### 2.2 2D Superlattice Materials with Different Band Alignment Modes

Based on the band alignment modes which drive electrons to migrate from a more negative conduction band to a less negative conduction band, whereas holes migrate from a more positive valence band to a less positive valence band, the 2D/2D superlattice materials can be classified into three types.

**Figure 3:**
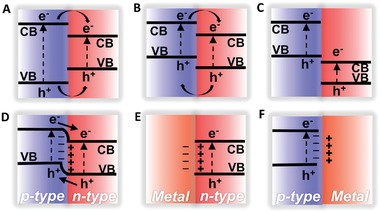
Band structure alignments and semiconductor types of 2D superlattice materials. A) Type I, B) Type II, C) Type III, D) p‐n heterojunction, E) metal/n‐type contacts, F) metal/p‐type contacts.

Type I—straddling gap band alignment (**Figure** **3**A): in this type of alignment, the conduction band edge of semiconductor A (SC‐A) is higher (more negative) than that of semiconductor B (SC‐B), whereas the valence band of SC‐A is lower (more positive) than that of SC‐B. Therefore, both the electrons and holes from SC‐A can be transported to the conduction band and valence band of SC‐B, respectively, which would not result in charge separation.^[^ ^123^, ^124^, ^125^ ^]^ Chen et al. proposed a 2D lateral hydrogenated‐silicene/halogenated‐silicene superlattice (S_H_S_X_ SL, X = F, Cl, Br, and I) with very large conduction band offset and small valence band offset.^[^ ^126^ ^]^ The band edge positions of S_H_S_X_ SL were accorded with the water‐splitting potential and the ability of absorbing light in the visible region, verifying that these superlattices are prospective for photo/electrocatalysis and optoelectronics devices.

Type II—staggered gap alignment: this type of alignment gives the best charge separation (Figure 3B), because the relative positions of the valence and conduction bands allow the mobility of electrons from the conduction band of SC‐A to that of SC‐B, whereas the holes move from the valence band of SC‐B to that of SC‐A. Z‐scheme is also one typical type II alignment, where the holes of SC‐A combine with the electrons of SC‐B at the interface and consequently make the electrons of SC‐A and holes of SC‐B available for photocatalytic activity.^[^ ^127^, ^128^, ^129^ ^]^ Most of the catalytic superlattice materials are in a type II configuration. Wei et al. proposed an in‐plane superlattice of MoS_2_/WS_2_ and studied by first‐principles calculations.^[^ ^130^ ^]^ Contributed by the type II—staggered gap alignment and the build‐in electric field, the in‐gap states were evoked by S vacancies and became more continuous in the superlattice MoS_2_/WS_2_ and the excitons could be spatially separated. In this superlattice structure, the mobility of carriers and the energy conversion efficiency could be greatly improved, and thus disclose enormous potential for solar cells and (photo)catalysts.^[^ ^130^ ^]^

Type III is called as broken gap band alignment, in which the relative positions of valence and conduction bands of SC‐A and SC‐B do not favor the transport of both electron and holes in either direction, and thus no charge separation are permitted, which have been rarely studied as catalytic materials (Figure 3C).^[^ ^131^ ^]^

### 2.3 2D Superlattice Materials with Different Semiconductor Types

Based on the type of semiconductors involved, the 2D/2D superlattices can simply be classified as p–p (between two p‐type semiconductors), n–n (between two n‐type semiconductors), and p–n (one p‐type and one n‐type semiconductor) heterojunctions (Figure 3D). In isotype heterojunctions (p–p and n–n junction), majority charge carriers could infuse between the two semiconductors with different band gaps, leading to detached charge carriers and modulated electronic configurations at the heterointerfaces by their disparate Fermi energy, which is of great significance to optimize the catalytic activity.^[^ ^132^ ^]^

p–n junction is an extremely efficient strategy toward electronic modulation and has drawn wide attention. In the p–n junctions, a potential difference at the interface can be generated without an exterior bias, which can restrain the electron and hole combination and boost the migration of electron and hole via a synergetic effect roused by the internal electric field and the band orientation of heterointerface.^[^ ^133^, ^134^ ^]^ Generally, the electrons tend to diffuse through the heterointerface of p–n junction from the n‐type semiconductor into the p‐type semiconductor, while the holes move in an opposite direction until the system achieves the Fermi energy balance, which can improve the catalytic performance with the strong oxidation of holes. The p–n junction usually has excellent optical characteristics benefited from the difference in electron affinity and favorable band gap alignment between the two semiconductors, which has been widely applied in solar cells and photo/electrocatalysis.^[^ ^133^, ^135^, ^136^, ^137^, ^138^ ^]^

Furthermore, n‐type and p‐type semiconductors can also be used to achieve rectifying junctions with metals (Figure 3E,F). Based on the electron transfer and superior electronic interaction properties caused by a Schottky effect at the semiconductor–metal interfaces, the heterogenous structures can display highly enhanced physicochemical/electrochemical reactivity, which not only are a pivotal part in some optoelectronic and electronic devices, but also in high‐performance catalysts. The changes of the electron density of metal centers and the corresponding band bending of the semiconductors triggered by Schottky junctions determine the carrier/charge transport involved into the electrochemical catalysis processes and thus govern the final catalytic activity.

### 2.4 2D Superlattice Materials with Different Constitutional Compositions

In terms of the constitutional materials, currently the main components for constituting 2D superlattices are semiconducting materials and group IV–V monolayers, such as graphene,^[^ ^100^, ^101^, ^139^, ^140^ ^]^ phosphorene,^[^ ^141^ ^]^ etc., specifically, TMDs,^[^ ^81^, ^100^, ^103^, ^106^, ^142^, ^143^, ^144^ ^]^ TMOs,^[^ ^140^, ^145^ ^]^ LDHs,^[^ ^80^, ^81^, ^82^, ^99^, ^101^, ^104^, ^105^, ^146^, ^147^, ^148^, ^149^, ^150^, ^151^ ^]^ etc.

The excellent electrical conductivity, thermal conductivity, and chemical affinity of graphene makes it widely studied and applied in the fields of catalysis, energy storage, optoelectronic devices, etc.^[^ ^152^, ^153^, ^154^ ^]^ The 2D materials with relatively weak electrical conductivity (e.g., MoS_2_, TMOs, LDHs, MXenes) are usually combined with graphene or reduced graphene oxide (rGO) to form 2D superlattices (MoS_2_/graphene,^[^ ^100^, ^139^ ^]^ TMOs/graphene,^[^ ^140^, ^145^, ^155^ ^]^ LDHs/graphene,^[^ ^80^, ^82^, ^101^, ^104^, ^105^, ^147^, ^148^, ^149^, ^150^ ^]^ MXene/graphene,^[^ ^156^ ^]^ etc.) to achieve excellent electrical conductivity for promoting the charge transfer and thus enhancing the electrocatalytic activity of the counter materials. In addition, graphene fabricated by chemical methods has abundant functional groups, strong hydrophilicity, and rich defects, which benefit to increase the number of active sites and enhance the electrocatalytic activity of 2D superlattices.

As another typical class of 2D materials, TMDs possess the characteristics of adjustable band gap and tailorable physicochemical properties, which can be used as ideal materials for energy storage, electrocatalysts, and electronic devices. The TMDs‐based superlattices mainly include LDHs/TMDs,^[^ ^81^ ^]^ graphene/TMDs,^[^ ^100^, ^139^ ^]^ g‐C_3_N_4_/TMDs,^[^ ^103^ ^]^ TMDs/TMDs,^[^ ^106^, ^142^, ^143^, ^144^ ^]^ TMOs/TMSs,^[^ ^81^ ^]^ etc.

The LDHs (or TMOs) are adopted to design highly stabile, high‐performance, and inexpensive catalysts for their controllable surface charging, regulable compositions, and large specific surface areas. For LDHs‐based superlattices, such as TMOs/LDHs,^[^ ^99^, ^146^ ^]^ TMDs/LDHs,^[^ ^81^ ^]^ graphene/LDHs,^[^ ^80^, ^82^, ^101^, ^104^, ^105^, ^147^, ^148^, ^149^, ^150^ ^]^ organic matters/LDHs,^[^ ^151^ ^]^ most of them are used as highly efficient electrocatalysts toward OER. Considering that TMOs usually have low electric conductivity, TMOs thus are usually combined with conductive materials to form 2D superlattices, such as Ti_0.87_O_2_/PDDA‐graphene,^[^ ^140^ ^]^ SrMnO_3_/LaMnO_3_,^[^ ^157^ ^]^ Ti_0.67_Fe_0.3_O_2_/rGO,^[^ ^155^ ^]^ Nb_3_O_8_/rGO,^[^ ^145^ ^]^ etc.

To date, over 70 kinds of MXene phases have been reported and the number is continuously growing, enabling this class of materials to be promising constitutional substrates for building 2D superlattice structures.^[^ ^110^, ^156^, ^158^, ^159^, ^160^ ^]^ Specifically, the extraordinary electric/electrochemical properties of MXenes provide them to be a good platform to develop new 2D MXene‐based superlattices for energy storage and conversion, such as multi‐low‐dimensional GerMXene superlattice heterostructures.^[^ ^161^ ^]^

Besides the above‐mentioned typical classes of 2D superlattice materials, with the development of intercalation and other techniques, 2D superlattices composed of organic molecules have brought into novel inorganic–organic hybrid superlattice structures, such as THAB/In_2_Se_3_,^[^ ^162^ ^]^ TiS_2_(HA)_0.025_,^[^ ^163^ ^]^ LDHs/Fe‐PP,^[^ ^164^ ^]^ polyaniline‐V_2_O_5_,^[^ ^165^ ^]^ etc. The appearance of new fabrication technologies and novel construction materials will provide further opportunities to design more 2D superlattice structures with desired properties for diverse applications.

## 3 Construction of 2D Superlattice Materials

The specific physical and chemical properties of these 2D materials lead to different approaches for the fabrication of 2D structures.^[^ ^166^ ^]^ For the 2D materials with weak interlayer bond force (e.g., graphene),^[^ ^167^ ^]^ a top‐down approach is more suitable.^[^ ^101^ ^]^ Single dispersed 2D monolayer nanosheets can be obtained by exfoliating the bulk materials, and then the corresponding superlattice materials can be obtained through layer‐by‐layer (LBL) assembly^[^ ^139^, ^151^, ^157^, ^164^, ^168^, ^169^ ^]^ or flocculation precipitation method.^[^ ^81^, ^82^, ^99^, ^100^, ^101^, ^104^, ^105^, ^110^, ^140^, ^145^, ^146^, ^149^, ^150^, ^155^, ^170^ ^]^ For the 2D materials with strong interlayer bond force (e.g., TMDs), a bottom‐up approach can reach high‐quality 2D superlattice structures.^[^ ^106^, ^142^, ^143^, ^144^ ^]^ For vertically stacking superlattice structures, single layer or multilayer 2D nanosheet A is first prepared, and then another single layer or multilayer nanosheet B is prepared on the surface of nanosheet A. By repeating this process for several times, high‐quality 2D superlattice materials with good crystallization can be obtained.^[^ ^143^, ^144^ ^]^ For the superlattice structure consisting of organic/inorganic hybrid materials, the intercalation and polymerization of organic molecules at the interlayers of the inorganic 2D materials should be employed.^[^ ^162^, ^163^, ^164^, ^165^ ^]^ Therefore, by looking at the physical and chemical properties of various constituting materials, appropriate methods can be selected to construct 2D superlattice materials, in return, to enhance the performance of the 2D materials. Some commonly employed fabrication methods for 2D superlattice structures are summarized in following sections.

### 3.1 Chemical Vapor Deposition (CVD)

Due to accurate control on the experimental parameters, the CVD method, a bottom‐up approach to grow materials on substrates from gaseous precursors, has been regarded as an effective way to synthesize high‐quality 2D materials, in which their heterostructures with precisely controlled thickness and layers can be achieved.^[^ ^106^, ^142^, ^171^ ^]^ For the CVD growth of 2D superlattice materials, the first 2D structure is deposited onto the substrate, and then the second 2D material grows either laterally surrounding or vertically stacking onto the first 2D material.

**Figure 4:**
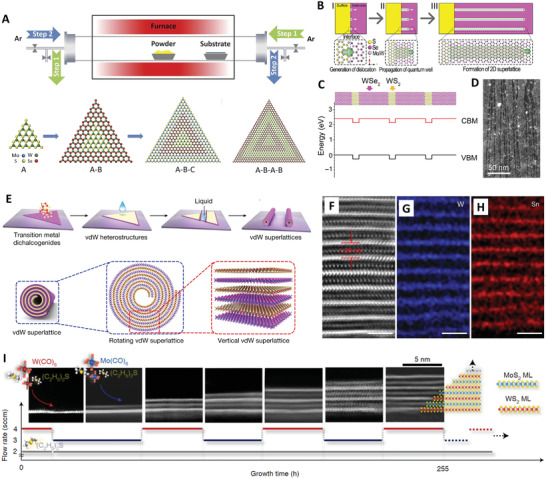
CVD growth of 2D superlattice materials. A) Schematic illustration of modified CVD epitaxial growth of lateral heterostructures. Reproduced with permission.^[^ ^106^ ^]^ Copyright 2017, American Association for the Advancement of Science. B) Schematic of the formation of lateral WSe_2_/WS_2_ superlattice. C) Schematic of atomic structural model and band structure alignment of the WSe_2_/WS_2_ lateral superlattice. D) Low‐magnification scanning transmission electron microscopy angular dark field (STEM‐ADF) image of the superlattice formation. Reproduced with permission.^[^ ^173^ ^]^ Copyright 2018, American Association for the Advancement of Science. E) Schematic view of the formation of roll‐up vdW superlattices. F–H) Cross‐sectional STEM image and corresponding EDS mapping of G) W and H) Sn of the SnS_2_/WSe_2_ vdW superlattice. Reproduced with permission.^[^ ^107^ ^]^ Copyright 2021, Springer Nature. I) The growth of MoS_2_/WS_2_ superlattices and corresponding high‐angle annular dark field (HAADF)‐STEM images. Reproduced with permission.^[^ ^144^ ^]^ Copyright 2021, Springer Nature.

The growth of vertically stacking 2D superlattice materials with multiple layers is very straightforward, which can be performed via a sequential growth process to repeat the deposition for several times. Unfortunately, due to the unexpected growth thermodynamic fluctuation and the inhomogenous nucleation associated with the 2D crystal growth, some undesired defects can exist within the prepared heterostructures, which sometimes bring some side effects into the devices constructed from these heterostructured superlattice materials. Furthermore, during the CVD growth, some unexpected impurities can form between the layers.^[^ ^142^, ^172^ ^]^ Zhang et al. developed a modified CVD method to prepare the lateral WS_2_‐WSe_2_‐WS_2_‐WSe_2_‐WS_2_ superlattice.^[^ ^106^ ^]^ During the synthesis, both sides of the silica tube were equipped with a gas inlet and a gas outlet to ensure the formation of a reverse flow from the substrate to the source for reducing the exposure to the high temperature and minimizing the thermal degradation, as shown in **Figure** **4**A. During the temperature‐swing stage, the continual cold argon flush was reversely inlet into the tube to cool the grown 2D crystals, to lower the thermal degradation, and to prevent the uncontrollable homogenous nucleation, and subsequently, to allow the growth of the WS_2_‐WSe_2_ lateral superlattice block‐by‐block. The lateral 2D heterostructured superlattice materials with good lattice orientation between two layers have been utilized to form p–n junctions with efficient photogenerated charge‐carrier transport and separation properties and been applied in photo‐response‐adjustable photovoltaic cells.^[^ ^142^, ^172^ ^]^ Recently, Zhou et al. developed a novel CVD approach for growing quantum wells within monolayer WSe_2_ (or MoSe_2_) nanosheets and then forming 2D WSe_2_ (or MoSe_2_)/WS_2_ (or MoS_2_) superlattice (Figure 4B). The growing process was modulated via individual misfit dislocations generated at the heterointerface of WSe_2_ (or MoSe_2_) and WS_2_ (or MoS_2_). The insertion of S and metal atoms into the dislocation cores caused the dislocation climb, and the Se atoms selectively substituted by S atoms were activated by the local strain field, which led to the WS_2_ (MoS_2_) quantum wells embedding into the WSe_2_ (MoSe_2_) monolayers to form periodic superlattice (Figure 4C,D).^[^ ^173^ ^]^ Furthermore, Zhao et al. reported a straightforward method to synthesize high‐order vertical superlattices via rolling up vdW heterostructures obtained by a CVD method, which was driven by the capillary force (Figure 4E).^[^ ^107^ ^]^ The scanning transmission electron microscopy (STEM) and energy dispersive X‐ray analysis (EDX) mapping images confirmed the formation of uniform vdW superlattices (Figure 4F–H).

To date, many types of 2D materials, such as nitrides,^[^ ^174^, ^175^ ^]^ carbides,^[^ ^176^, ^177^ ^]^ oxides,^[^ ^178^, ^179^ ^]^ etc., and their heterostructures,^[^ ^180^, ^181^ ^]^ have been successfully prepared under CVD conditions. It should be noted that the CVD growth method needs only very simple equipment to reach precisely controllable experimental parameters, such as temperature, atmosphere, and pressure, which enable CVD to be the most widely employed fabrication method for the growth of 2D and other thin‐film materials.^[^ ^182^ ^]^ This is also the only method that can realize the growth of laterally aligned 2D superlattice structures. Even though high‐quality 2D superlattice materials with excellent performance have been achieved by the CVD method, some demands on the experimental conditions, including highly pure and expensive precursors, high‐quality substrates, accurately controllable temperatures and pressures, strict flow directions, difficulties on large size and scale production, and so on, have hindered the large‐scale production via this approach. In addition, the energy consumption and the cost of the CVD technique are also significant concerns for its engineering production of advanced materials.^[^ ^110^ ^]^

The metal organic chemical vapor deposition (MOCVD) method can be employ for contrasting 2D superlattice by using metal‐organics with higher equilibrium vapor pressures as precursors, e.g., W(CO)_6_, Mo(CO)_6,_ C_4_H_10_S, etc. The reaction temperature of MOCVD is usually lower than that of the conventional CVD.^[^ ^183^ ^]^ Jin et al. reported an atomic epitaxial growth method for preparing the 2D superlattice with two or more kinds of dissimilar TMDs monolayers (Figure 4I). Kinetics‐controlled growth behavior can be achieved in the near‐equilibrium limit by MOCVD, which is crucial for monolayer‐by‐monolayer stacking.^[^ ^144^ ^]^ In addition, Kumar et al. reported a vdW superlattice composing of alternating layers of TMDs and dielectric insulators, which realized near‐unity absorption and maintained enhanced opto‐electronic properties and photoluminescence emission.^[^ ^143^ ^]^ However, some challenges yet exist in the MOCVD method, such as the rapid decomposition of the prepared superlattice structures and the contamination of amorphous carbon give rise to low crystal quality as well as slow growth rate, which should be solved in future studies for improving the quality and efficiency.^[^ ^163^, ^164^ ^]^

### 3.2 Layer‐by‐Layer (LBL) Assembly in Solution

**Figure 5:**
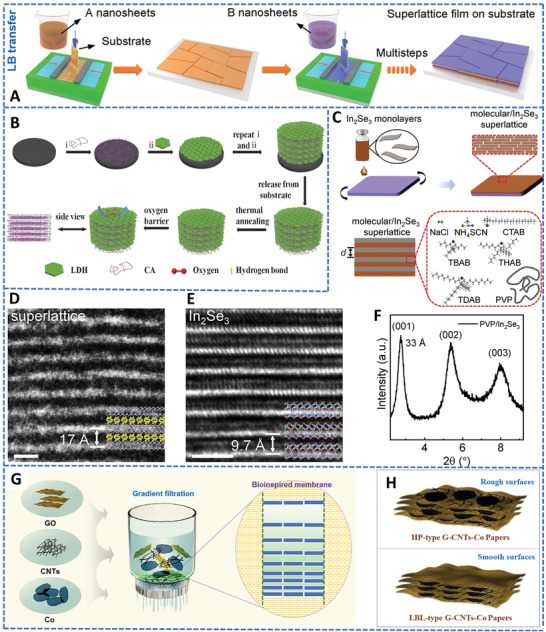
LBL assembly of 2D superlattice materials. A) Schematic illustration of solution phase LBL assembly of 2D superlattice materials. Reproduced with permission.^[^ ^114^ ^]^ Copyright 2020, Wiley‐VCH. B) Schematic illustration of the spin‐coating LBL assembly of (CA/LDHs)*_n_* films. Reproduced with permission.^[^ ^151^ ^]^ Copyright 2014, Wiley‐VCH. C) Schematic representation for the formation of THAB/In_2_Se_3_ organic/inorganic superlattices via a spin‐coating LBL assembly. D) Cross‐sectional HRTEM images of THAB/In_2_Se_3_ superlattice. E) THAB/In_2_Se_3_ superlattice after annealing at 400 °C. Scale bars: 2 nm. F) XRD patterns of THAB/In_2_Se_3_ superlattices after annealing. Reproduced with permission.^[^ ^162^ ^]^ Copyright 2021, Elsevier. G) Schematic illustration of construction of bamboo‐membrane‐bioinspired Co_3_O_4_/graphene heterostructures though a vacuum filtration LBL approach. H) Schematic illustration of the surface states of the HP‐type membrane fabricated from the mixing solution and the LBL‐type membrane. Reproduced with permission.^[^ ^91^ ^]^ Copyright 2021, Wiley‐VCH.

The LBL deposition method is a thin‐film fabrication technique, by which heterostructures form by depositing alternating layers of materials with opposite charges and applying wash steps in between, via immersion, spin, spray, fluidics, electrophoresis, etc.^[^ ^112^, ^114^ ^]^ This method is very convenient for the fabrication of 2D superlattice materials by repeatedly depositing the 2D building blocks with oppositely charged onto the substrate, because the 2D nanosheets can be deposited flatly on substrates. The LBL method can also accurately regulate the components and structures of the superlattices. In a typical LBL assembly, as shown in **Figure** **5**A, the substrate is first immersed into the suspension of A nanosheets for several minutes and washed to remove excess solution from the substrate, and then, the substrate is immersed in another colloidal nanosheet solution and washed again. By repeating the steps to reach the desired layers or thickness, the 2D superlattice materials can be obtained.^[^ ^114^ ^]^

During the LBL process, two pivotal aspects affect the quality of the 2D superlattice materials: i) the suspensions for the LBL assembly should be a stable dispersion of the well‐exfoliated 2D nanosheets; ii) the starting 2D nanomaterials should be oppositely charged on their surfaces and terminated with affiliative surface groups to allow the perfect deposition of nanosheets by layers without wrinkles.^[^ ^114^, ^184^ ^]^ Li et al. prepared 2D superlattice materials by alternately stacking Ti_0.91_O_2_ or Ca_2_Nb_3_O_10_ oxide nanosheets with Mg_2/3_Al_1/3_(OH)_2_ LDHs nanosheets by alternately immersing the substrate into the two 2D nanosheets suspensions.^[^ ^185^ ^]^ The oxide and LDH nanosheets dispersed into formamide solutions were alternately deposited onto a Si wafer substrates to form densely stacked superlattice. After assembly, the layer distance of 1.2 and 2.0 nm was observed, which was consistent with the summation of the thickness of the LDH nanosheets and the corresponding oxide nanosheets, Ti_0.91_O_2_ and Ca_2_Nb_3_O_10_, respectively. It should be noted that the superlattice structure prepared via the LBL method is not perfectly stacked and there are often some overlaps and gaps between the sheets.^[^ ^182^ ^]^

Besides the immersion of substrates into solutions, spray coating or spin coating has also been employed for LBL assembly.^[^ ^151^, ^186^ ^]^ The LBL assembly realized by coating techniques can dramatically reduce the interlayer defects existing in the solution immersion method, owing to that an intense spray force or centrifugal force during the deposition can accelerate the diffusion of the solvent between the layers, exclude the impurities, agglomerates, micelles, gaseous bubbles, emulsions, etc., from the interlayer space, and eliminate the wrinkles of the nanosheets, and thus improve the quality of the superlattice.^[^ ^187^ ^]^ Dou et al. fabricated a superlatticed material composed of LDH nanoplatelets and cellulose acetate (CA) by commutative spin‐coating and a followed thermal annealing treatment (Figure 5B).^[^ ^151^ ^]^ The as‐prepared film displayed optical transparency, high flexibility, and compositional uniformity, owing to the homogenous layered architecture constituting by well‐dispersed LDH nanosheets inside the CA matrix. Vacuum filtration provides another approach for the LBL assembly of 2D layered superlattice structure. Lin et al. dispersed the exfoliated In_2_Se_3_ monolayers in a dimethyl formamide solvent for synthesizing ordered 2D molecular hybrid superlattices with alternating inorganic 2D monolayers and organic molecular layers by spin coating (Figure 5C). The corresponding high‐resolution transmission electron microscopy (HRTEM, Figure 5D,E) and X‐ray diffraction (XRD, Figure 5F) verified the superlattices with a period of ≈1.7 nm.^[^ ^162^ ^]^ In addition, Mei et al. fabricated a layered superlattice structure by alternately depositing the highly dispersed 2D Co_2_O_3_ and graphene nanosheets via the vacuum filtration method (Figure 5G).^[^ ^91^ ^]^ To enhance the interlayer electronic transport properties, 1D carbon nanotubes were also introduced into the layers. By repeating the depositions, a bamboo‐membrane‐inspired layered structure with tailored interlayer spaces were fabricated as the free‐standing anode for Li‐ion batteries (Figure 5H).

From the above representative examples, the LBL assembly method possesses some apparent advantages in the fabrication of 2D superlattice materials, such as simple equipment, easy operation, good control on layers and thickness, and high‐quality products when combined with other coating techniques. This method, unsurprisingly, has also some challenges in the fabrication of 2D superlattice materials. For example, it needs high‐quality and well‐dispersed 2D nanosheet suspensions as starting materials. During the fabrication, it is difficult to fully avoid the existence of interlayer defects resulted by the overlap or gap of the 2D nanosheets. Furthermore, the repeating assembly is a time‐consuming process and also difficult to reach thick films. Therefore, further optimization is needed to achieve high‐quality superlattices with a scaling‐up production potential.

### 3.3 Molecular Intercalation

2D/2D inorganic–organic hybrid structures have led to the development of many new superlattice materials.^[^ ^112^ ^]^ One of straightforward method for preparing 2D organic–inorganic superlattices is to insert the polymer molecules directly into the interlayers of the inorganic layered host materials.^[^ ^78^ ^]^ Due to the existence of interlayer space within the layered inorganic materials, some small molecules can penetrate into the layered structure and expand the lamellae spacing, and further allow the insertion of large organic molecules into the interlayers of the 2D materials to form 2D inorganic–organic superlattice materials.^[^ ^188^ ^]^

**Figure 6:**
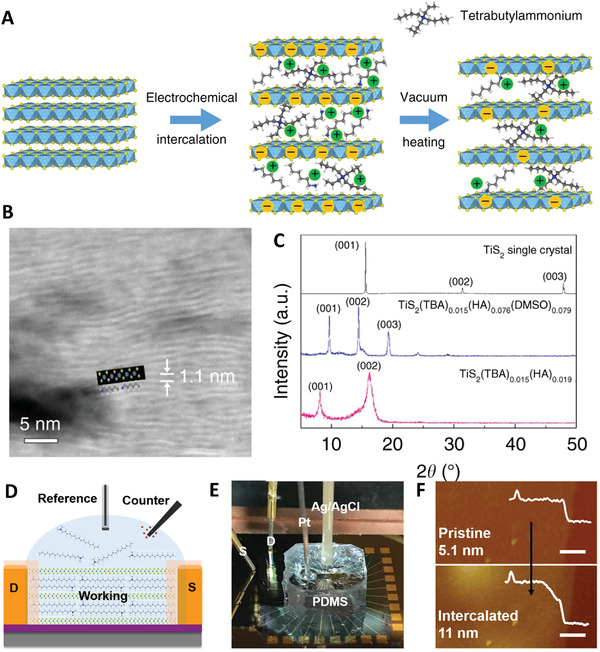
Molecule intercalation for 2D superlattice materials fabrication. A) Schematic illustration of 2D superlattice consisting of TiS_2_ and HA/TBA molecules via a combination of electrochemical intercalation and vacuum heating. B) HAADF‐STEM image of TiS_2_(TBA)_0.013_(HA)_0.019_. C) XRD patterns of TiS_2_, TiS_2_(TBA)_0.015_(HA)_0.074_(DMSO)_0.079_ and TiS_2_(TBA)_0.013_(HA)_0.019_. Reproduced with permission.^[^ ^163^ ^]^ Copyright 2017, Springer nature. D) Schematic illustration of the devices for in situ monitoring the molecule intercalation process. E) Optical photograph of the in situ molecule intercalation apparatus. F) AFM measurements of the thickness of MoS_2_ before and after CTAB intercalation. Scale bars: 500 nm. Reproduced with permission.^[^ ^190^ ^]^ Copyright 2019, American Chemical Society.

Via the electrochemical intercalation method, hexylammonium and tetrabutylammonium molecules were intercalated into layered TiS_2_ to form a TiS_2_[tetrabutylammonium]*_x_* [hexylammonium]*_y_* superlattice structure (**Figure** **6**A), in which the concentration of tetrabutylammonium and hexylammonium could be controlled by temperature, as the hexylammonium molecules had a lower boiling temperature than that of the tetrabutylammonium. The HRTEM image of TiS_2_[tetrabutylammonium]*_x_* [hexylammonium]*_y_* suggested the organic layers and inorganic layers stacked alternatively with a 1.1 nm interlayer distance (Figure 6B), which was well matched with XRD patterns (Figure 6C).^[^ ^163^ ^]^ Similarly, a covalent PbBDT (BDT = 1,4‐benzenedithiolate) organic–inorganic hybrid superlattice was fabricated by electrochemically intercalating BDT molecules into layered PbS_2_.^[^ ^189^ ^]^ Moreover, He et al. systematically studied the electrochemical molecular intercalation method for preparing 2D organic–inorganic superlattice materials (Figure 6D,E).^[^ ^190^ ^]^ Atomic force microscope (AFM) images showed the thickness change of MoS_2_ nanosheet before (5.1 nm) and after (11 nm) cetyltrimethylammonium bromide (CTAB) intercalation (Figure 6F). They designed an in situ electronic and optical characterization apparatus to detect the intermediate and monitor the dynamic evolution of superlattice materials. In a typical case, it was found that the intercalation of CTAB into the interlayer of MoS_2_ could lead to the phase transformation from 2H‐MoS_2_ to 1T‐MoS_2_. Furthermore, this work demonstrated that the molecular intercalation process could be extended to a variety of 2D layer materials, such as graphene, TiS_2_, ReS_2_, WSe_2_, and PdSe_2_. In this electrochemical intercalation method, the insertion size can be adjusted according to the interlayer spacing of the host materials.^[^ ^190^ ^]^ The most important factor in the organic molecule intercalation is the polymerization of monomers within the host structures. Thus, the selection of specific host materials (such as V_2_O_5_^[^ ^166^, ^191^ ^]^) and the induced electric energy often play critical roles in driving the intercalation and the polymerization processes during the electrochemical molecular intercalation of 2D inorganic–organic superlattice materials.^[^ ^190^, ^192^ ^]^

The development of 2D organic–inorganic superlattice materials via the electrochemical intercalation further expands the family of 2D materials, increases the versatility of superlattice materials, and provides a new platform to trigger some new functions that are not present in existing materials. This method only needs common electrochemical devices and demonstrates its high efficiency in obtaining high‐quality products. However, the intercalation of molecules is only applicable to the layered host materials with large interlayer spacing and weak interlayer bonding forces. At present, the intercalation of 2D materials mainly focuses on undersized organic molecules. The insertion of macromolecules is yet a challenge. Furthermore, only vertically stacked 2D superlattice structures can be fabricated via this approach. Therefore, further study on this topic is necessary for fabricating more generalized inorganic–organic hybrid 2D superlattice materials.

### 3.4 Liquid Phase Flocculation/Precipitation Method

**Figure 7:**
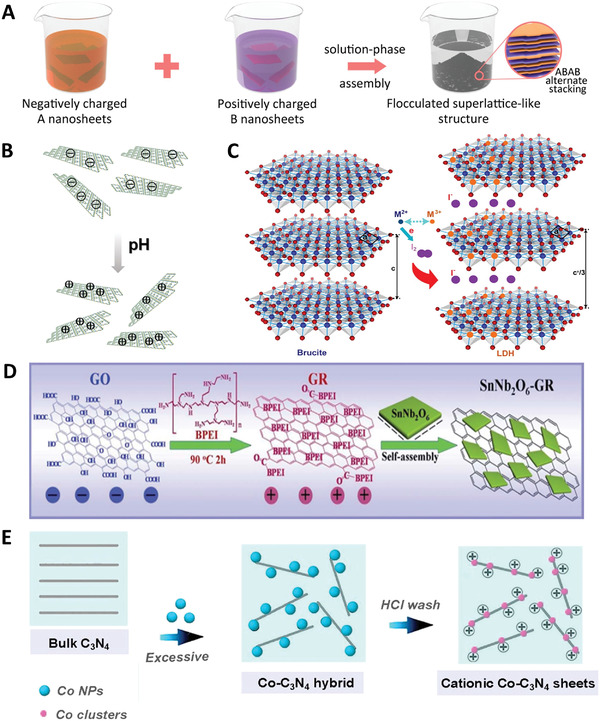
Liquid phase flocculation/precipitation preparation of 2D superlattice materials. A) Schematic illustration of the formation of superlattice via liquid phase flocculation/precipitation. Reproduced with permission.^[^ ^79^ ^]^ Copyright 2019, American Chemical Society. B) Schematic illustration of the surface charging regulation via pH value controlling. C) Topochemical oxidation for tailoring the positive charge of M^3+^ against M^2+^ in LDHs. Reproduced with permission.^[^ ^195^ ^]^ Copyright 2012, American Chemical Society. D) BPEI polycation‐modified graphene for constructing SnNb_2_O_6_‐GR superlattice. Reproduced with permission.^[^ ^197^ ^]^ Copyright 2014, The Royal Society of Chemistry. E) Schematic diagram of Co mental nanoclusters for modifying the surface charge of the 2D C_3_N_4_ nanosheets. Reproduced with permission.^[^ ^201^ ^]^ Copyright 2018, American Chemical Society.

The liquid phase flocculation or precipitation method shares a similar principle with the LBL assembly method, in which oppositely charged surfaces of the constitutional nanosheets are first modified and the electrostatic force‐driven self‐assembly then occurs when two suspensions are mixed together (**Figure** **7**A). Different to the LBL assembly of superlattice, the self‐assembly of the superlattice takes place at the same time during the liquid flocculation or precipitation processes, which allows the fabrication of 2D superlattice materials at a high yield and high efficiency. This method thus is a feasible way for large‐scale fabrication of superlattice structures for possible engineering applications.

During the liquid flocculation or precipitation synthesis, the most critical step is to regulate the surface charge of the constitutional 2D monolayer nanosheets. To achieve electrostatic force‐induced assembly, the surfaces of the two constitutional nanosheets are needed to be modified into different charges, which can make the two oppositely charged nanosheets to be attracted and stacked alternately with each other to form a 2D superlattice in a liquid environment, while the nanosheets with same charges cannot be attached to form agglomerates. Thus, the surface charges are critical to form well‐ordered alternating 2D superlattice structure. The major approaches on regulating the surface charges of the nanosheets are summarized as follows:

i) *pH value adjustment*: it has been well studied that the surface potential (*ζ*‐potential) of the particles in a suspension solution varies as the change of pH values, as a response to the ionic equilibrium between the solid nanoparticle surface and the solvent environment.^[^ ^193^, ^194^ ^]^ In a solution, the surface atoms of the exfoliated nanosheets react with the solvent, e.g., a hydrolysis reaction with water, to form exposed surface groups, and finally reach a dynamic equilibrium. This dynamic equilibrium, usually a result of competition between the acidic sites and the alkaline sites, however, is very sensitive to the ionic environment, specifically the variation of pH values. For example, in the cases of ternary oxide and carbides, Y_2_Si_2_O_7_ and Ti_3_AlC_2_, the ionic equilibrium can be elucidated from the individual ion reaction equilibrium and the ion distribution diagram as a function of pH.^[^ ^193^, ^194^ ^]^ Therefore, the surface charges of the two constitutional 2D nanosheets can be regulated into opposite by adjusting the pH value of the solution (Figure 7B). Kwon et al. fabricated a g‐C_3_N_4_/MoS_2_ superlattice structure via the liquid flocculation.^[^ ^103^ ^]^ The g‐C_3_N_4_ was fabricated by melamine thermal polycondensation and then adjusted into a cationic state (+32 mV) by titrating the pH value to 3. At that pH value, the exfoliated MoS_2_ nanosheets showed a negative *ξ*‐potential of −35 mV. By mixing these two nanosheets with opposite charges together, an electrostatic force‐driven self‐assembly happened to form the 2D g‐C_3_N_4_/MoS_2_ superlattice structure.
ii) *Topochemical oxidation of cations*: It has been found that an oxidative intercalation based on redoxable transition metal elements, such as Fe, Co, Ni, etc., led to the structural transformation from a brucite structure (e.g., Co_1−_ *_x_* Fe*_x_* (OH)_2_, Co(OH)_2_, and Co^2+^_1−_ *_x_* Ni^2+^ *_x_* (OH)_2_) to an LDH structure, accompanied with the partial oxidation of divalent transition metal elements into trivalent state by oxidizing agents, such as iodine (I_2_) or bromine (Br_2_) (Figure 7C).^[^ ^195^, ^196^ ^]^ The LDH nanosheets after exfoliation are positively charged and repel with each other to form stable suspensions. Via this principle, positively charged CoNi LDHs were synthesized by the topochemical oxidation method by using bromine as the oxidant, as shown in the following equation^[^ ^196^ ^]^
(1) $$\begin{array}{ccc} & & {\mathrm{Co}}_{1-x}^{2+}{\mathrm{Ni}}_{x}^{2+}{\left({\mathrm{OH}}^{-}\right)}_{2}+1/6{\mathrm{Br}}_{2}+0.5H_{2}O\\ & & \;\to \left[{\left({\mathrm{Co}}_{1-x}^{2+}{\mathrm{Ni}}_{3x/2}^{2+}\right)}_{2/3}{\mathrm{Co}}_{1/3}^{3+}{\left({\mathrm{OH}}^{-}\right)}_{2}\right]\left[{\mathrm{Br}}_{1/3}^{-}\cdot 0.5H_{2}O\right] \end{array}$$

During oxidation, the halogen ions (Br^−^, I^−^) were intercalated into the LDHs layers to expand the interlayer spacing for further invading of electrolyte and intercalating molecules. After exfoliation, the anions between the layers of LDHs would lose, resulting in the positive charge of the monolayer LDHs nanosheets.

iii) *Polycations or polyanions modification*: To modify the surface charge of the 2D nanosheets into opposite charging, the adsorption of polycations and polyanions onto the surfaces is another effective way. As shown in Figure 7D, the GO with a negative charge can be tuned into a positive charge by grafting with branched polyethylenimine (BPEI) molecules,^[^ ^197^ ^]^ in which a strong interaction occurs between the negative oxygen‐containing functional groups on GO nanosheets and the abundant positive amino groups of BPEI. By modifying with BPEI, the surface charge was tailored from −60 to +50 mV, which allowed the adsorption with negatively charged SnNbO_5_ to form a 2D SnNb_2_O_6_ nanosheet‐graphene (SnNb_2_O_6_‐GR) heterostructure. Similarly, Cai et al. reported that various anionic nanosheets, such as GO, Ti_0.87_O_2_^0.52−^, and Ca_2_Nb_3_O_10_^−^, could be modified into positively charged surfaces by using polycation PEI molecules, which could be then used for the fabrication of superlattice structures, such as a 2D rGO‐Ti_0.87_O_2_^0.52−^ superlattice by mixing PEI‐GO with the Ti_0.87_O_2_^0.52–^ suspension followed by annealing at 300 °C.^[^ ^198^ ^]^ In another case, Chu et al. used a polycationic solution as the medium for the ball milling of h‐BN, graphite, MoS_2_, and other layered materials to obtain cationic 2D nanosheets.^[^ ^199^ ^]^

There are some advantages for the polycation or polyanion modification of surface charges. First, the polycation or polyanion modification molecules can be easily removed from the superlattice structures by annealing. Second, in suspension solutions, the polymer molecules, such as PEI and poly(acrylic acid), can provide steric force to stabilize the nanosheet suspension without flocculation for a long time, which can eliminate the defects within the 2D superlattice structure resulted by the self‐stacking or curling of the nanosheets.^[^ ^193^, ^194^ ^]^ Third, the long‐chain polymer molecules adsorbed on the surface of the 2D nanosheets can suppress the oxidation of some oxygen‐sensitive 2D materials.

iv) *Metal nanoclusters modification*: Metal nanoclusters have been proven to act the role of functional groups to effectively adjust the surface charge of 2D materials in an aqueous solution.^[^ ^200^ ^]^ Sun et al. manipulated the surface electronic properties of 2D C_3_N_4_ through the loading of a small amount of Co clusters on to the 2D nanosheets (<5 wt%) (Figure 7E). Due to the different electronegative properties, the N atoms in the 2D C_3_N_4_ are chemically affinitive with the Co nanoclusters to form a 2D Co‐C_3_N_4_ nanostructure, which can tune the negatively charged C_3_N_4_ surfaces into positively charged Co‐C_3_N_4_ surfaces.^[^ ^201^ ^]^ Similarly, Fe nanoclusters have also been used to modify 2D C_3_N_4_ into a positively charged 2D Fe‐N‐C structure, which were then assembled with the negatively charged MXene to form a 2D Fe–N–C/MXene superlattice structure for ORR electrocatalysis.^[^ ^110^ ^]^

The metal nanoclusters modification method can effectively modify the surface charge as the polyanion or polycation reagents but avoid the loss of the conductivity and charge transport behaviors of the materials. Furthermore, via this method, catalytically active sites can be simultaneously introduced into the superlattice structures for achieving enhanced chemical activity, enabling it to be a promising fabrication method for the preparation of 2D superlattice electrocatalysts.

## 4 Application of 2D Superlattice in Electrocatalysis

Electrocatalytic reactions are necessary steps in some emerging sustainable energy technologies, such as water splitting,^[^ ^81^, ^101^ ^]^ fuel cells,^[^ ^110^, ^202^ ^]^ metal–air batteries,^[^ ^99^, ^203^ ^]^ etc. The high‐efficient electrocatalysts adopted in the electrodes of these energy devices can significantly reduce the higher energy barrier of the involved electrochemical reactions in the systems.^[^ ^204^, ^205^ ^]^ Compared with other 2D electrocatalysts, the 2D heterostructured superlattice materials possess high specific heterointerfaces, which offer some tailorable interfacial couplings to regulate the interfacial charge and mass transport properties and the electronic structures, which can provide ultrafast interfacial transport to accelerate the catalytic reaction and optimize the electronic configurations for thermoneutral intermediates adsorptions during the electrocatalytic processes.

**Figure 8:**
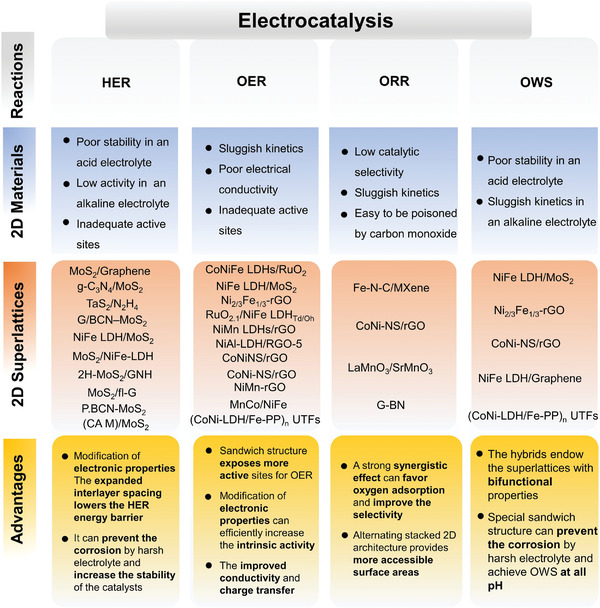
Advantages of 2D superlattice materials for electrocatalytic applications.

Rational design of the heterostructured 2D superlattices with high catalytic capability and environmental durability by using nonmetallic or transition metal 2D materials offers a promising pathway toward high‐efficiency electrocatalysis. First, the periodic stacking of the 2D superlattice structures allows the full exposure of the active sites on each nanosheet to the reactants. Second, the hybridization of two property‐complementary nanosheets at a molecular level provides the opportunity in regulating the charge/carrier transport and separation behaviors through proper band structure alignment, and thus improves the electron mobility and distribution for enhancing the catalytic performance.^[^ ^79^, ^81^, ^100^ ^]^ Third, the sandwiched superlattice structure can provide protection to the environmental vulnerable material inside the layers from the external environment impact or the corrosion, and thus increase the stability and environmental durability of the catalysts. Fourth, the hybridization of two components at the molecular level can effectively compensate for the shortcomings of a single component and can realize multifunctional catalysis. In this section, the recent progress in 2D superlattice materials as efficient electrocatalysts for HER, OER, OWS, and ORR is summarized. **Table** **1** summarizes some representative examples of 2D superlattice materials in electrocatalysis applications together with the corresponding mechanisms. Some unique advantages of the 2D superlattice materials in electrocatalysis are summarized in **Figure** **8**.

**Table 1:** Representative research progress of 2D superlattice materials for electrocatalysis in a recent decade

	Electrocatalytic performance				
2D superlattice materials	Reaction	Electrolyte	(Over) Potential at 10 mA cm^−2^	Tafel slope	Activity origin or mechanisms
MoS_2_/Graphene [100]	HER	0.5 m H_2_SO_4_	137 mV	48.7 mV dec^−1^	The intensive charge transfer in the superlattices (MoS_2_/graphene)offers a rapid kinetics property. Highly expanded interlayer spacing lowers the Gibbs free energy of HER reactants.
NiFe‐LDHs/MoS_2_ (NiAl‐LDHs/MoS_2_) [81]	OER	1 m KOH	250 mV	45 mV dec^−1^	The valence band edge of MoS_2_ is located at lower energy than the conduction band edge of LDHs, the charge transfer will occur from the LDHs to MoS_2_, and thus the materials have significantly enhanced affinity with HER or OER reactants.
	HER	0.5 m H_2_SO_4_	180 mV	82 mV dec^−1^	
Co_2/3_Ni_1/3_ NS/GO [80]	OER	1 m KOH	259 mV	35.7 mV dec^−1^	The introduction of Ni atoms into the Co hydroxide promotes the oxidation of Co(II) for favorable adsorption/desorption of the reactant (−OH) and/or the intermediates (such as −O*), which changes the rate controlling step into the formation of OOH* (O∗ + OH^−^ → OOH* + e^−^).
RuO_2.1_/NiFe LDH_Td/Oh_ [168]	OER	1 m KOH	205 mV	55 mV dec^−1^	Excellent conductive nanolayers and interfacial electronic coupling contribute to fast electron transport and desired adsorption of OER intermediates.
g‐C_3_N_4_/MoS_2_ [103]	HER	0.5 m H_2_SO_4_	232 mV	89.4 mV dec^−1^	Superlattice structure with high active site exposure. Charge transfer from g‐C_3_N_4_ to MoS_2_ makes extra enhancement for HER due to the increases in electron density of MoS_2_, which would accelerate the HER kinetics of the rate‐determining Volmer step in an acidic medium.
MSGR [213]	HER	1 m KOH	47 mV	52 mV dec^−1^	The intensive interfacial interaction of hybrids improves the structural ordering of electrocatalysts and accelerates the charge transfer. The special superlattice structure restrains the repacking of 2D nanosheets; which facilitates the mass diffusion kinetics.
Gate modulated (CA·M)/MoS_2_ [230]	HER	0.5 m H_2_SO_4_	14 mV	21 mV dec^−1^	Hydrogen‐bonded CA·M could be used for activating the electron density of MoS_2_. The corresponding electron transfer happens from CA·M to MoS_2_ enhances the HER activity.
Ru moiré superlattices [216]	HER	1 m KOH	24 mV	33.8 mV dec^−1^	The strain effect induced by twisting causes downward shift of the d‐band center and the lattice contraction, which is crucial to weakened the H* adsorption and improve HER performance.
WS_2_ moiré superlattices [215]	HER	0.5 m H_2_SO_4_	60 mV	40 mV dec^−1^	The strain effect on WS_2_ superlattices activates the basal plane by altering the electronic configuration and the active centers of the W‐edge and S‐edge, which can largely decrease Δ_GH*_.
2D‐PtND/LDHs [224]	HER	1 m KOH	25 mV	32.2 mV dec^−1^	The flat structure of the 2D‐PtND and the exclusively exposed {110} crystal led to intensive electronic coupling effect with NiFe LDHs laminates.
MnCo‐O/NiFe‐OH [146]	OER	1 m KOH	233 mV	46 mV dec^−1^	The projected density of state suggests a slightly positive shift for Ni d band center after being assembled into a superlattice, which could adjust adsorption properties of intermediates during the OER process.
G‐BN [228]	ORR	Potassium phosphate buffer solution at pH = 7	*E*_1/2_ = 0.79 V	67 mV dec^−1^	The G‐BN superlattice expedites charge transfer and maximizes its electronic structure for transferring electrons to oxygen, which may arise from the appropriate electron transfer due to the modification of the electronic structure of G by BN.
Fe‐N‐C/MXene [110]	ORR	0.1 m KOH	*E*_1/2_ = 0.84 V	‐	Alternately stacked 2D architecture provides more accessible surface areas. A strong synergistic effect between Fe‐N‐C and MXene can facilitate electronic modulation for favorable oxygen adsorption.

### 4.1 Water‐Splitting Catalysis

Electrochemical water splitting (H_2_O = H_2_ + O_2_) includes two half‐reactions: HER at the cathode and OER at the anode. The theoretical splitting voltage of water is 1.23 V. However, the voltage applied to trigger the actual reaction is much higher than 1.23 V, due to the polarization of the electrode. This voltage beyond the theoretical value is called as overpotential.^[^ ^206^ ^]^ The periodically stacked 2D superlattice materials for water‐splitting catalysis can reduce the overpotential of the reactions by optimizing the intermediate adsorption and enhancing the charge and mass transport along the heterostructured interfaces via effective electronic coupling between the two different nanosheets. Via proper structure alignment and materials selection, advanced 2D superlattice catalysts can achieve low‐overpotential OWS with high energy efficiency.^[^ ^79^, ^81^, ^101^, ^103^, ^207^ ^]^

#### 4.1.1 HER

HER is a two‐electron process as the cathodic half‐reaction of water electrolysis for the generation of hydrogen gas. There are two reaction processes of HER in the electrolyte. First, H^+^ (in an acid environment) or H_2_O molecule (in an alkaline or neutral environment) is adsorbed onto the surface of the catalyst to form an adsorbed hydrogen atom (H*) (Volmer reaction). Then, hydrogen (H_2_) forms by combining H^+^ or H_2_O with the $H_{\mathrm{ads}}^{*}$ via the Heyrovsky reaction, or two adsorbed hydrogen atoms $H_{\mathrm{ads}}^{*}$ react directly to form H_2_ via the Tafel process. If H_2_ forms via the Heyrovsky reaction, the catalytic mechanism is usually called as the Volmer–Heyrovsky mechanism, and if H_2_ forms via a Tafel reaction, it is called as a Volmer–Tafel process.

The HER process is mainly governed by the adsorption of free energy of hydrogen intermediate species.^[^ ^206^ ^]^ MoS_2_ has been calculated as a very promising catalyst for HER in an acidic medium, due to its favorable hydrogen intermediate adsorption energy at its edges, and it is expected to replace the Pt‐based electrocatalyst.^[^ ^208^ ^]^ However, MoS_2_ suffers from its insufficient HER activity, owing to its inferior electrical conductivity and less exposed active sites.^[^ ^209^, ^210^ ^]^ It is fortunate that the design of heterostructures by combing the 2D TMDs nanosheets with other 2D materials, such as graphene and TiO_2_ nanosheets, can provide much enhanced H_2_ generation catalytic activity, due to the formation of specific interfacial electronic coupling for favorable intermediate adsorption.^[^ ^95^, ^211^ ^]^

**Figure 9:**
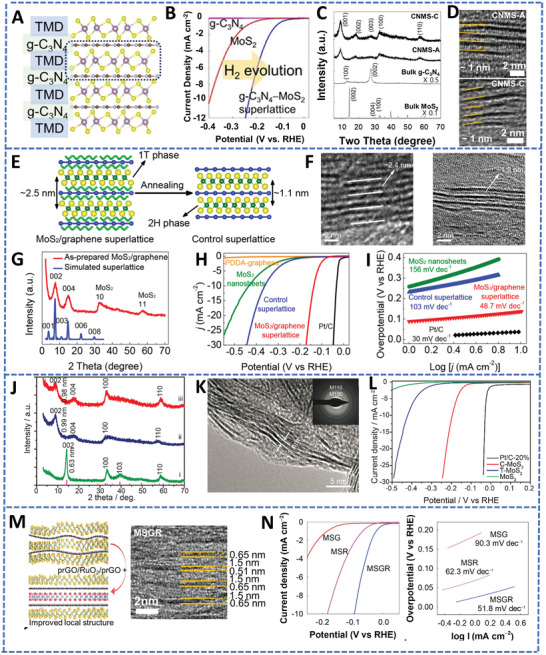
2D superlattice materials for HER. A) Structural diagram of TMD/g‐C_3_N_4_ superlattice, B) the corresponding HER polarization curves, C) the corresponding XRD patterns, and D) the HRTEM images. Reproduced with permission.^[^ ^103^ ^]^ Copyright 2020, Elsevier. E) Structural diagram of MoS_2_/graphene superlattice, and the corresponding F) HRTEM images, G) XRD patterns, H) polarization curves, and I) Tafel slopes. Reproduced with permission.^[^ ^100^ ^]^ Copyright 2018, American Chemical Society. C‐MoS_2_ superlattice for HER catalysis: J) the XRD patterns, K) the HRTEM image, and L) the HER polarization curves. Reproduced with permission.^[^ ^212^ ^]^ Copyright 2018, American Chemical Society. M) Schematic illustration and corresponding HRTEM image of trilayer prGO/RuO_2_/prGO superlattice (MSGR), N) the corresponding HER polarization curves and Tafel slopes, comparing with binary MoS_2_−prGO (MSG) or MoS_2_−pRuO_2_ (MSR) superlattices. Reproduced under the terms of the Creative Commons CC‐BY license.^[^ ^213^ ^]^ Copyright 2022, The Authors. Published by Wiley‐VCH.

A 2D MoS_2_/g‐C_3_N_4_ superlattice was developed for high‐efficiency HER performance (**Figure** **9**A,B).^[^ ^103^ ^]^ As shown in the XRD pattern (Figure 9C) and HRTEM characterization (Figure 9D), the hybridization of g‐C_3_N_4_ and MoS_2_ displayed well‐distributed layer spacing for effective electrolyte diffusion. With the interstratification of g‐C_3_N_4_ and MoS_2_, the strong interfacial electronic coupling, and the formed nitrogen vacancies, bifunctionality and enhanced electrochemical HER catalysis performance were achieved. This 2D MoS_2_/g‐C_3_N_4_ superlattice structure only needed 231 mV to reach 10 mA cm^−2^ in an acid medium, while MoS_2_ needed 384 mV under the same condition (Figure 9B).

One critical factor for efficient HER is the conductivity of the catalysts. Therefore, 1T‐MoS_2_ with metallic properties is a more desirable candidate for HER electrocatalysis.^[^ ^100^ ^]^ Combining the metallic 1T‐MoS_2_ with the conductive graphene to form a MoS_2_/graphene superlattice (Figure 9E), the conductivity of the catalyst system was improved and the charge transfer properties were promoted. The HRTEM image of the MoS_2_/graphene superlattice (Figure 9F) showed a periodic multilayer structure with a periodic spacing of 2.4 nm, which was twice of the spacing than the control MoS_2_/graphene superlattice annealed at 300 °C in Ar (Figure 9G). The MoS_2_/graphene superlattice exhibited enhanced electrocatalytic HER activity with a low overpotential of 137 mV (Figure 9H) and a Tafel slope of 73 mV dec^−1^ (Figure 9I), compared with the overpotential of 435 mV to reach 10 mA cm^−2^ for the MoS_2_ nanosheets.^[^ ^100^ ^]^ The 1T‐MoS_2_ has good electroconductibility and catalytic activity, while the 2H‐MoS_2_ has poor electrical conductivity but a thermodynamically stable phase. Ma et al. synthesized the periodic superlattice based on 2H‐MoS_2_ and graphitic nanocarbon hybrid (C‐MoS_2_) based on a hydrothermal threonic acid‐intercalated 2H‐MoS_2_ and a subsequent graphitization process.^[^ ^212^ ^]^ XRD pattern and HRTEM confirmed the superlatticed structure of C‐MoS_2_ (Figure 9J,K). The conducting unilaminar graphitic nanocarbon promoted the electron transfer and shortened the diffusion distance. This sandwich‐like superlattice (C‐MoS_2_) also demonstrated much improved electrocatalytic activity and durability toward HER (Figure 9L). Furthermore, Kwon et al. prepared a trilayer prGO/RuO_2_/prGO superlattice (MSGR) through the LBL deposition method (Figure 9M). The intensive interfacial interaction of MSGR improved the structural ordering of the electrocatalysts and accelerated the charge transfer properties. Furthermore, the pores formed during the restacking of the superlattice nanosheets facilitated the mass diffusion kinetics. Thus, the MSGR showed excellent activity as an effective HER electrocatalyst (Figure 9N).^[^ ^213^ ^]^

**Figure 10:**
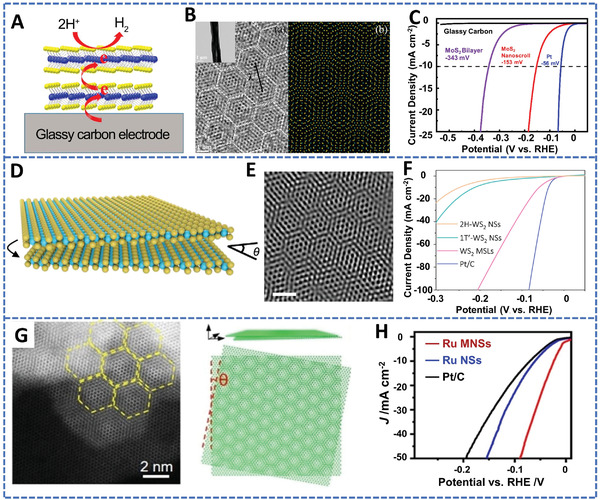
2D moiré superlattice materials for HER. MoS_2_ moiré superlattice structure: A) Structural diagram, B) corresponding HRTEM image, and C) HER polarization curves. Reproduced with permission.^[^ ^211^ ^]^ Copyright 2019, American Chemical Society. WS_2_ moiré superlattice structure: D) Structural diagram, E) corresponding HRTEM image, and F) HER polarization curves. Reproduced with permission.^[^ ^215^ ^]^ Copyright 2021, Springer Nature. Ru multilayered nanosheet moiré superlattice: G) HRTEM image and corresponding structural diagram, H) HER polarization curves. Reproduced with permission.^[^ ^216^ ^]^ Copyright 2022, Wiley‐VCH.

Moiré superlattices with peculiar physicochemical property have received wide attention very recently. Yuan et al. synthesized a MoS_2_ moiré superlattice (**Figure** **10**A,B) with a twisted structure.^[^ ^214^ ^]^ Due to the depressed interlayer barriers, charges could transfer readily from the electroconductive substrate to the active centers in a moiré superlattice, resulting in excellent HER performance. As shown in Figure 10C, the MoS_2_ moiré superlattice presented a low overpotential of 137 mV to reach 10 mA cm^−2^ in a 0.5 m H_2_SO_4_ solution. Afterward, Xie et al. prepared a twisted WS_2_ moiré superlattice electrocatalyst via a hydrothermal method, the structural diagram and the corresponding HRTEM image are shown in Figure 10D,E. The strain effect in the WS_2_ superlattices activated the basal plane with altering the electronic configuration and the active centers of the W‐edge and S‐edge, which largely decreased the Δ*G*_H*_. Meanwhile, the superaerophobic and superhydrophilic characteristics further improved the HER activity, leading to a low overpotential of 60 mV to reach 10 mA cm^−2^ (Figure 10F).^[^ ^215^ ^]^

Based on above mentioned examples, the 2D TMDs‐based superlattices can possess altered electronic structures and tailored interlayer spacing to achieve better HER activity. The dilated interlamellar spacing exposes more unsaturated S atoms and edge active sites, which provides adequate space for the hydrogen intermediate species transport. However, the 2D TMDs‐based superlattices are effective for acid HER catalysis, but they are yet very sluggish in alkaline and neutral aqueous electrolytes. The development of novel 2D superlattices for effective HER catalysis in neutral and alkaline electrolytes should be another urgent task in this research field.

In addition to TMDs, Zhang et al. prepared a Ru moiré superlattice structure based on Ru multilayer nanosheets via a facile wet‐chemical method (Figure 10G).^[^ ^216^ ^]^ The strain effect induced by twisting caused downward shift of the d‐band center and the lattice contraction, which are crucial to weaken the H* adsorption and improve the HER performance. The overpotential of Ru moiré superlattice was 24 mV at 10 mA cm^−2^ for the alkaline HER (Figure 10H).

Therefore, the interlayer potential barrier existed in the 2D heterostructures is crucial for HER catalysis, which can be activated when the electrons surmount the interlayer potential barriers and transfer from the conducting substrates to the active centers. The proper construction of 2D superlattice materials can effectively reduce this interlayer potential barrier and boost the electron transfer during the catalytic reactions. In addition, some exfoliated 2D monolayers (such as MoS_2_) are highly unstable and intend to aggregate/restack during applications, the construction of 2D superlattice structure is an effective strategy to prevent the agglomeration of individual 2D structures. At least, the 2D superlattices can only form loosely restacked agglomerates, due to the existence of separation structures between the 2D materials, which still provide highly open interlayer spacing and accessible active centers for high‐performance catalysis, endorsing them to be a promising class of HER catalysts.

#### 4.1.2 OER

OER is the anodic reaction of water electrolysis. OER process needs to go through a complex multistep four‐electron reaction, in which the breaking of the H—O—H bond and the formation of O—O are kinetically sluggish processes. The reaction mechanisms are different in acid conditions and alkaline conditions in terms of the generation of adsorbed OH* intermediate and the release of O_2_.^[^ ^205^, ^217^ ^]^ Generally, in the first step, H_2_O or OH^−^ reacts with the active sites of the catalyst surface to form adsorbed *OH, which then combines with H_2_O or OH^−^ to generate O*. In a following step, two O* combines to form oxygen (O_2_) together with another possibility of O* combines with H_2_O or OH^−^ to form *OOH and then further be oxidized into O_2_.^[^ ^218^ ^]^

LDHs have attracted extensive attention as a class of excellent OER catalysts for their low overpotentials compared to other metal oxide catalysts, contributed by the existence of cations with mixed valences for modulable coordination environment and synergistic interaction.^[^ ^219^ ^]^ However, the conductivity and the leaching of active elements into the solution of LDHs are still needed to be further addressed for better OER performance.

**Figure 11:**
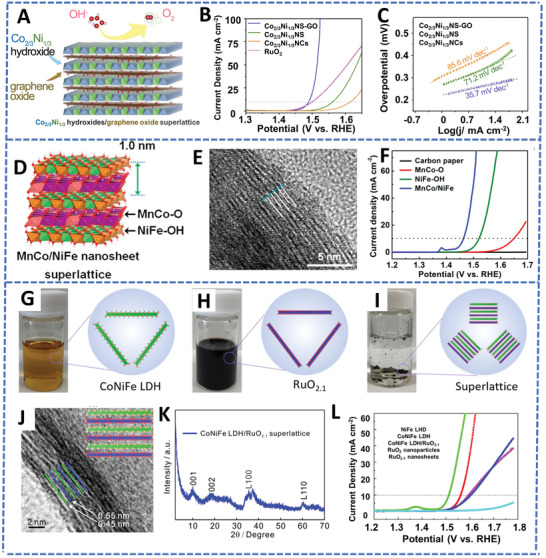
2D superlattice materials for OER. Co_2/3_Ni_1/3_ NS/GO superlattice structure: A) Structural diagram, B) OER polarization curves, and C) Tafel slopes. Reproduced with permission.^[^ ^80^ ^]^ Copyright 2019, Wiley‐VCH. MnCo‐O/NiFe‐OH superlattice structure: D) Structural diagram, E) HRTEM image, and F) OER polarization curves. Reproduced with permission.^[^ ^146^ ^]^ Copyright 2022, American Chemical Society. CoNiFe LDHs/RuO_2.1_ superlattice: G) Optical photographs of CoNiFe LDHs, H) RuO_2.1_ nanosheets dispersions, and I) CoNiFe LDHs/RuO_2.1_ superlattice. J) HRTEM image, K) XRD pattern, L) OER polarization curves. Reproduced with permission.^[^ ^99^ ^]^ Copyright 2020, American Chemical Society.

The CoNi LDHs^[^ ^80^ ^]^ and NiMn LDHs^[^ ^104^ ^]^ were employed to construct periodical nanostructures with graphene or rGO for addressing the disadvantages of insufficient efficiency of the electrocatalysts with single active component.^[^ ^80^ ^]^ As shown in **Figure** **11**A–C, the formation of dual metallic hydroxide and graphene oxide superlattice presented enhanced electron transport and more exposed active sites, leading to a highly efficient OER reaction with a low overpotential of 259 mV to reach 10 mA cm^−2^ and a small Tafel slope of 35.7 mV dec^−1^. It revealed that the introduction of Ni atoms into the Co hydroxide promoted the oxidation of Co(II) for favorable adsorption/desorption of the reactant (−OH) and/or the intermediates (such as −O), which changed the rate‐controlling step into the formation of OOH* (O^∗^ + OH^−^ → OOH* + e^−^). Moreover, the heterointerface of CoNi LDHs/GO superlattice optimized the adsorption of intermediates (HO*, O*, HOO*) and improved the electron transfer during the catalysis. Sasaki et al. synthesized NiMn/(r)GO superlattice by using NiMn LDHs and GO/rGO nanosheets.^[^ ^104^ ^]^ The NiMn/rGO achieved a low OER overpotential of 260 mV and a small Tafel slope of 46 mV dec^−1^. Afterward, NiAl‐LDHs/RGO‐5^[^ ^149^ ^]^ was also explored for high‐efficient OER electrocatalysis. Unlike the superlattices mentioned earlier, Zhang et al designed a noncarbon TMOs/LDHs superlattice structure consisting of MnCo‐O and NiFe‐OH with an interlayer space of ≈1 nm (Figure 11D,E),^[^ ^146^ ^]^ which presented a small overpotential (233 mV) and excellent long‐term electrocatalytic durability (Figure 11F), ascribed by the significantly improved conductivity and electrocatalytic activity of the interfaces of the superlattice structure. In addition, Dong et al. employed a solvent evaporation method to prepare a c‐oriented NiFe‐LDHs‐F superlattice from the colloidal 2D NiFe‐LDH nanosheets. The self‐assembled NiFe‐LDHs‐F showed a superior OER activity (about 220 mV of overpotential at 10 mA cm^−2^).^[^ ^220^ ^]^ Recently, the more robust RuO_2_ with high electrocatalytic activity was used to replace the role of carbon materials in the superlattice structures. By combining the trimetallic LDHs with RuO_2.1_ nanosheets, a CoNiFe LDHs/RuO_2.1_ 2D superlattice (Figure 11I–K) was fabricated by mixing the colloidal solution of positively charged LDHs (Figure 11G) with the negatively charged RuO_2.1_ nanosheets (Figure 11H). The 2D CoNiFe LDHs/RuO_2.1_ superlattice structure presented an improved OER performance with an overpotential of 281 mV to reach 10 mA cm^−2^ in 1 m KOH (Figure 11L). The turnover frequency value of the CoNiFe LDHs/RuO_2.1_ superlattice at an overpotential of 300 mV was about 0.045 s^−1^. The excellent performance of this superlattice was benefited from a strong interfacial electronic coupling between the constitutional nanosheets, which provided better electric conductivity and suppressed the side reactions existing in traditional OER catalysts.^[^ ^99^ ^]^

The alternating stacking manners at a molecular scale of the LDHs‐based superlattices enable a direct interfacial connection between two different nanosheets. This unique stacking behavior greatly shortens diffusion distance and exhibits excellent oxygen evolution activity. However, the composition changes resulted by leaching and surface structure reconstruction during the OER catalysis could dramatically alter the surface active sites and the real reaction centers, and the associated influence on the catalytic activity have attracted increasing attention.^[^ ^221^ ^]^ In fact, most of the OER electrocatalysts are working as precatalysts, such as 2D transition metal materials, including transitional metal oxides/hydroxides/phosphide/carbides/nitrides, TMDs, etc.^[73,^ ^222^, ^223^ ^]^ The variations of surface structures and compositions could also happen in 2D superlattices, which should be operando monitored during the OER catalysis. The mechanical understanding on the transition states of OER catalysts may help the design of real phase and structure of 2D superlattices toward OER.

#### 4.1.3 Overall Water Splitting

**Figure 12:**
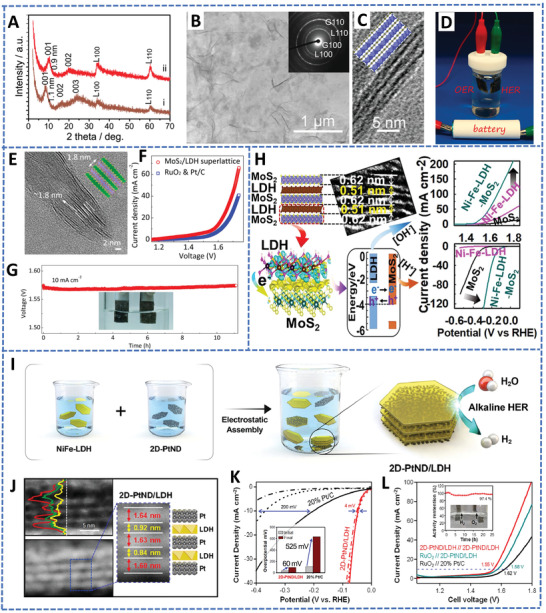
2D superlattice materials for OWS. Ni_2/3_Fe_1/3_‐rGO superlattice: A) XRD pattern, B) TEM image, C) HRTEM image, and D) OWS electrolyzer driven by a 1.5 V AA battery. Reproduced with permission.^[^ ^101^ ^]^ Copyright 2015, American Chemical Society. MoS_2_/LDHs superlattice: E) HRTEM image, F) OWS polarization curve, and G) stability test. Reproduced with permission.^[^ ^79^ ^]^ Copyright 2019, American Chemical Society. NiFe LDHs/MoS_2_ superlattice: H) Structural diagram, HRTEM image, and HER and OER polarization curves. Reproduced with permission.^[^ ^81^ ^]^ Copyright 2018, American Chemical Society. 2D‐PtND/LDHs superlattice: I) Procedure for preparing Pt nanodendrite/LDHs. J) HRTEM image and corresponding structural diagram, K) HER polarization curves, and L) OWS polarization curves. Reproduced with permission.^[^ ^224^ ^]^ Copyright 2022, American Chemical Society.

Different to the half‐reactions, OWS needs a reasonable construction of bifunctional electrocatalyst for the low energy barrier reaction of both HER and OER. Ma et al synthesized a Ni_2/3_Fe_1/3_‐rGO superlattice via a homogenous precipitation method (**Figure** **12**A–C).^[^ ^101^ ^]^ In this superlattice structure, the high conductivity of rGO and the high intrinsic electrocatalysis of LDHs work together to reach excellent OWS performance. An OWS cell composed of Ni_2/3_Fe_1/3_‐rGO superlattice was driven by a single 1.5 V AA battery (Figure 12D). Whereafter, three different superlattices, NiFe‐LDHs/graphene, MoS_2_/graphene, and MoS_2_/NiFe LDHs, were fabricated from graphene, MoS_2_, and NiFe LDHs nanosheets (Figure 12E).^[^ ^79^ ^]^ The MoS_2_/LDHs superlattice exhibited much higher electrocatalytic performance and stability than those of NiFe‐LDHs/graphene and MoS_2_/graphene superlattices, only a potential of 1.57 V was needed to reach 10 mA cm^−2^ for OWS (Figure 12F,G). In the MoS_2_/LDHs superlattice, the LDHs offer rich active centers for OH^−^ intermediates adsorption, while MoS_2_ provides optimized adsorption of H^+^, leading to low energy OWS.

Currently, the 2D superlattice materials with both high HER and OER activity in a universal pH environment are still a big challenge. Figure 12H presents electrostatically assembled NiFe LDHs/MoS_2_ and NiAl LDHs/MoS_2_ superlattice structures for high‐efficient water splitting in an acid environment.^[^ ^81^ ^]^ The hybridization of LDHs with MoS_2_ distinctly enhanced the chemical affinity with OH^−^ and H^+^ and decreased the energy for the generation of OH* and H* intermediates, and thus boosted both the HER and the OER activity. The conduction band edge of MoS_2_ is located at a lower energy than the valence band edge of LDHs, which promoted the charge transfer kinetics from the LDHs to the MoS_2_ and effectively improved the separation of carriers. Specifically, the holes generated in LDHs improved the OH^−^ binding energy on the surface of the catalyst and reduced the OER overpotential. The interesterification with MoS_2_ layers also significantly improved the chemical stability of LDHs in acidic media and achieved long‐term stable catalysis for over 10 h. Therefore, the construction of LDHs/MoS_2_ superlattice with a strong interfacial electronic coupling provides bifunctional catalytic activity, improved electronic conductivity, enhanced acidic stability, remarkable charge transfer, and separation capability, and offers promising potential for low‐energy OWS.

The iron porphyrin (Fe‐PP) with a good electrical conductivity could enhance the electron transfer for LDHs. Based on this understanding, well‐ordered ultrathin film (UTF) (CoNi‐LDHs/Fe‐PP)*_n_* superlattice electrodes were fabricated from layered double hydroxide nanosheets (LDH NSs) and iron porphyrin (Fe‐PP) though an electrostatic LBL method.^[^ ^164^ ^]^ This work emphasized that the appropriate thickness of the periodic film was significant for OWS, an excess thickness would confine the electron transfer and affect the electrocatalysis performance.

Hydroxides can also combine with noble metals to form bifunctional superlattice. Hong et al. developed a 2D‐Pt/NiFe‐LDHs superlattice consisting of 2D Pt nanosheets and NiFe‐LDHs for OWS (Figure 12I,J). The charge‐relocated interfacial bond between Pt and Ni(OH)_2_ had an intensive impact on the electronic structure of the construction materials and the sandwich structure creates abundant additional active centers at the interfaces. This unique combination gave rise to an HER mass activity of 11.21 times higher than that of Pt/C (Figure 12K). On the other hand, the incorporation of Pt into the superlattice offers the NiFe‐LDHs with distinguished OER performance. Contributed by the merits from both components, the 2D‐PtND/LDHs||2D‐PtND/LDHs electrolyzer only needed 1.55 V to reach 10 mA cm^−2^ for OWS in an alkaline medium (Figure 12L).^[^ ^224^ ^]^

Due to majority electrocatalysts are only active for either HER or OER, the 2D superlattices allowing heterostructuring/hybridizing of the components with different properties is a powerful way to promote the performance of the existing catalysts and generate new properties based on the strong synergistic effects for offering efficient dual or multifunctional catalytic activity for OWS. For industrial application, the standard 10 mA cm^−2^ current density for OWS is not enough to meet the practical requirements. The development of bifunctional electrocatalysts based on 2D superlattices to reach ampere‐level current density should be a future task. Moreover, the long‐term durability and stability at large current density are also big problems. The understanding of composition and structure reconstruction behavior and the design of environmental durable 2D superlattice‐based catalysts are highly desired to reach the expectations for practical applications.

### 4.2 ORR Catalysis for Fuel Cells or Metal–Air Batteries

ORR is the cathodic reaction of fuel cells or metal–air batteries and has complex reaction mechanisms, involving multiple charge transfer steps and elementary reactions with different reaction intermediates.^[^ ^225^ ^]^ The ideal fuel cell cathodic reaction is the full reduction of oxygen, which is a four‐electron process. However, the reduction potential of the four‐electron reaction is higher than that of the two‐electron process, and the dissociation energy of the O—O bond in oxygen is higher than that of the formation of H_2_O_2_.^[^ ^226^, ^227^ ^]^ The two‐electron process usually thus occurs with the production of H_2_O_2_, which causes damage to the proton exchange membrane. As a result, it is significant to develop highly effective catalysts for promoting the ORR with a four‐electron process.

**Figure 13:**
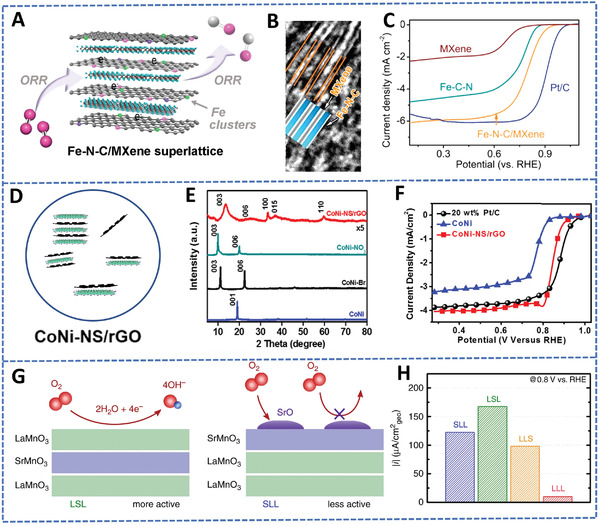
2D superlattice materials for ORR. Fe‐N‐C/MXene superlattice: A) Structural diagram, B) HRTEM image, C) ORR polarization curves. Reproduced with permission.^[^ ^110^ ^]^ Copyright 2020, American Chemical Society. CoNi LDH/rGO superlattice structure: D) Structural diagram, E) XRD pattern, and F) ORR polarization curves. Reproduced with permission.^[^ ^147^ ^]^ Copyright 2019, Elsevier. (LaMnO_3_)_2_/(SrMnO_3_) superlattice: G) Structural diagram, H) ORR performance at 0.8 V. Reproduced with permission.^[^ ^157^ ^]^ Copyright 2018, Springer Nature.

On this account, Jiang et al. fabricated a 2D Fe‐N‐C/MXene superlattice with 5.2% Fe loading by a liquid flocculent method (**Figure** **13**A,B).^[^ ^110^ ^]^ The obtained Fe‐N‐C/MXene superlattice used as an ORR catalyst showed a positive initial potential of 0.92 V, a half‐wave potential of 0.84 V (Figure 13C), and high‐stability in the alkaline electrolyte, which endorse it to be a promising candidate to replace the noble metal catalysts. A strong synergistic effect between the Fe‐N‐C and the MXene modulated the electronic structure for a favorable oxygen adsorption. It was suggested that the alternately stacked 2D architecture provided more accessible surface areas to drive a four‐electron transfer pathway, to maintain excellent long‐term stability, and to allow further modification at its surface or interfaces.

Some 2D materials, such as LDHs, are not favorable for the four‐electron process involved catalysis. The steric effect of LDHs can prevent the dissociation of hydrogen peroxide and the decomposition of O—O bond, which are not expected for the four‐electron pathway of ORR.^[^ ^228^ ^]^ Wang et al. proposed a superlattice structure based on CoNi LDH nanosheets and rGO for ORR electrocatalysis (Figure 13D,E).^[^ ^147^ ^]^ Inheriting the advantages of catalytic CoNi hydroxide nanosheets and electroconductive rGO, the CoNi LDHs/rGO superlattice exhibited a comparable performance to that of the commercial Pt/C and suppressed the peroxide yield to be <7% (Figure 13F). These results confirmed that the hybridization of LDHs with electroconductive graphene could effectively promote the dissociation of the O—O bond on the LDHs and thus effectively promote the four‐electron process.

Eom et al. synthesized a (LaMnO_3_)_2_/(SrMnO_3_) superlattice via molecular beam epitaxy.^[^ ^157^ ^]^ In this superlattice structure, placing the SrMnO3 layer not only modulated the Mn electronic configuration for more efficiently ORR but also eliminated the detrimental effect in reducing the surface site availability (Figure 13G,H). In addition, nonmetallic ORR electrocatalysts with superlattice structures have also been reported. For example, Primo et al. reported a graphene‐boron nitride superlattice (G‐BN). Due to the modification of the electronic structure of graphene by BN, the G‐BN superlattice expedited the charge transfer to oxygen, showing a superior electrocatalytic performance toward ORR.^[^ ^229^ ^]^

According to the progress achieved in the application of 2D superlattice materials for effective ORR catalysis, it is concluded that the combination of the components with different properties into the superlattices can achieve enhanced catalytic activity, accelerated catalytic kinetics, and much improved selectivity and stability. While the effectiveness of the 2D superlattice catalysts has been verified, the enhancement mechanisms and the roles of each component and the interfaces played in the catalytic processes yet need to be further studied and clarified with the help of some sophisticated technologies, such as advanced in situ and operando characterization techniques.

## 5 Summary and Prospects

In this review, we summarized the preparation methods of 2D superlattice materials and their applications in electrocatalysis (HER, OER, OWS, and ORR). Some commonly used fabrication methods, such as CVD, LBL assembly, molecular intercalation, and liquid phase flocculation/precipitation method, have been reviewed. The liquid phase flocculation/precipitation method allows the preparation of high‐quality 2D superlattice materials at a large‐scale by tuning the two constitutional 2D structures with opposite surface charges then assembling in a solution via electrostatic attraction.

The 2D superlattice materials have been employed as effective electrocatalysts for water‐splitting reactions or ORR, due to their unique structural advantages: i) the superlattice materials composed of two alternately stacks allows the full exposure of the catalytic active sites on different surfaces; ii) the intensive interfacial interaction between the alternately stacked nanosheets can modulate the electronic structure, promote charge separation and transfer properties, regulate the intermediate adsorption/desorption behaviors, and maximize the synergetic effect for electrocatalysis; iii) the superlattice materials can provide the coexistence of multifunctional sites on the surfaces to realize bifunctional or multifunctional catalysis or other Janus‐type properties or reactions; iv) the superlattice materials with proper selection of constitutional materials can provide durable environmental tolerance for long‐term operation under harsh or hazardous environments.

Although significant progress has been achieved in the discovery of 2D superlattice materials, some challenges and opportunities yet exist in the synthesis and application of this emerging type of materials.

i) While plenty of types of 2D superlattice materials have been synthesized for various applications, there is a vast space to design more heterostructured superlattice materials with unique chemical and physical properties. With the reports of novel 2D materials, such as 2D metal nanosheets,^[^ ^226^, ^231^ ^]^ black phosphorus,^[^ ^232^, ^233^ ^]^ 2D metal–organic frameworks,^[^ ^234^, ^235^ ^]^ 2D covalent organic frameworks, etc., more novel 2D superlattice materials are expected to be reported in near future.
ii) Although many feasible synthesis methods for 2D superlattice materials have been proposed, the efficient and low‐cost synthesis of 2D superlattice materials still needs to be further explored. CVD method allows the precise control of the stacking layers and stacking modes and provides high‐quality products with excellent purity and crystallinity. However, this method suffers from low yield and high energy consumption and is unfavorable for the industrial production of 2D superlattice materials. The liquid phase flocculation or precipitation method can realize high‐yield production of 2D superlattice materials in solution. The biggest challenge is that the contamination or surface adsorbed impurities bring unavoidable impurities or defects at the interfaces or on the surfaces of the materials. Therefore, it is necessary to develop advanced preparation technology for the preparation of 2D superlattice materials at a high quality and also high yield.
iii) The diverse options on the selection of the constituent materials for 2D superlattice materials provide abundant opportunities in tailoring the chemical and physical properties, but on the other hand, bring many difficulties in identifying the synergy mechanisms during the electrocatalysis. The complex surface and interface chemistry put further challenges in understanding the involved chemical reactions. Even though pretty a few experimental and theoretical investigations have been performed on the mechanism understanding, further efforts and new technologies are needed for the development of this class of materials. For example, the development of in situ*/*operando technologies, such as in situ X‐ray absorption fine structure, in situ differential electrochemical mass spectrometry, in situ Fourier transform infrared spectroscopy, in situ TEM, etc.,^[^ ^236^, ^237^ ^]^ will provide important support for exploring the intrinsic properties and the electrochemical reaction mechanisms of 2D superlattice materials.

It has been evidenced the development of 2D superlattice materials has provided new opportunities into the design of novel advanced materials and catalyst via their unique combination and configuration of 2D materials with similar or even opposite properties. This class of materials expands the family of 2D materials and opens a new way to further maximize the advantages of 2D materials. It is believed that more advanced 2D superlattice materials will be innovated and contributed to solving the real‐world challenges of the world.

## Conflict of Interest

The authors declare no conflict of interest.
